# How Early Veno-Venous Malformation Can Jeopardize a Perfect Kawashima Repair: A Compilation of Two Cases and Review of Literature

**DOI:** 10.7759/cureus.100260

**Published:** 2025-12-28

**Authors:** Sachin Talwar, Vishal V Bhende, Deepakkumar V Mehta, Purvi R Patel, Mahesh H Bhatt, Mathangi Krishnakumar, Amit Kumar, Gurpreet Panesar, Kunal A Soni, Kartik B Dhami, Nirja P Patel, Raag P Patel, Preeti P Raj, Ashwin S Sharma, Saptak P Mankad

**Affiliations:** 1 Cardiothoracic and Vascular Surgery, All India Institute of Medical Sciences, New Delhi, IND; 2 Pediatric Cardiac Surgery, Bhanubhai and Madhuben Patel Cardiac Centre, Shree Krishna Hospital, Bhaikaka University, Karamsad, IND; 3 Pediatric Cardiac Surgery, Sri Sathya Sai Sanjeevani Centre for Child Heart Care and Training in Pediatric Cardiac Skills, Navi Mumbai, IND; 4 Radiodiagnosis and Imaging, Pramukhswami Medical College, Shree Krishna Hospital, Bhaikaka University, Karamsad, IND; 5 Pediatrics, Pramukhswami Medical College, Shree Krishna Hospital, Bhaikaka University, Karamsad, IND; 6 Pediatric Interventional Cardiology, Bhanubhai and Madhuben Patel Cardiac Centre, Shree Krishna Hospital, Bhaikaka University, Karamsad, IND; 7 Anesthesiology, St John's Medical College Hospital, Bengaluru, IND; 8 Pediatric Cardiac Intensive Care, Bhanubhai and Madhuben Patel Cardiac Centre, Shree Krishna Hospital, Bhaikaka University, Karamsad, IND; 9 Cardiac Anesthesiology, Bhanubhai and Madhuben Patel Cardiac Centre, Shree Krishna Hospital, Bhaikaka University, Karamsad, IND; 10 Internal Medicine, Gujarat Cancer Society Medical College, Hospital and Research Centre, Ahmedabad, IND; 11 Internal Medicine, Dev Medical Hospital, Vadodara, IND

**Keywords:** cavopulmonary connection, congenital heart disease, interrupted inferior vena cava, kawashima operation, pulmonary arteriovenous malformation, veno-venous malformation

## Abstract

Systemic veno-venous malformation (VVM) often develops following a Kawashima operation (KO), a procedure that makes a connection between the superior vena cava (SVC) and the pulmonary artery (PA) in the setting of a blocked inferior vena cava (IVC), in the presence of complex cyanotic congenital heart disease (CCHD) of a single ventricle.

This case report describes two 12- to 13-month-old female patients weighing 6 kg who underwent the Kawashima operation. We found that VVM could be identified using cineradiography, above and below the diaphragm, following a Kawashima operation. We noted a significant decline in systemic oxygen saturation beginning on postoperative day 9 in one patient, temporally associated with angiographic identification of systemic VVMs, while the second patient maintained stable room-air saturations throughout follow-up despite similar hepatic venous incorporation. These findings suggest that targeted radiographic evaluation for systemic VVMs may be useful in selected patients with early postoperative desaturation following the Kawashima operation, enabling timely risk stratification and surveillance.

## Introduction

Veno-venous malformation (VVM) can cause marked hypoxemia following a Kawashima operation (KO) in patients presenting with a univentricular anomaly and interrupted IVC, which results in venous blood from the lower limb to the superior vena cava (SVC) through either the azygous vein or the hemiazygos vein, or both [1-4]. The incidence of interrupted inferior vena cava (IVC) is 1:5000. In almost 90% of patients with KO, it is a typical variant that is not associated with isomerism or fetal anomalies. In 0.07% and 8.7% of normal people, the IVC is persistent; however, it mostly doesn’t manifest any symptoms. Typically, IVC detection in adults is due to examination for other ailments. During embryological development, the hepatic and pre-renal segments fail to unite, which is the main reason for intra-hepatic interruption of the IVC along with azygos continuation. The actual prevalence of this condition is approximately 0.5%-2.1% among the general population. A hemizygous continuation that drains into the right atrium through a persistent left SVC is an uncommon occurrence.

Although systemic venous collateral formation has been described following cavopulmonary connections, including Glenn and Fontan circulations, Kawashima-specific data, particularly addressing early postoperative systemic VVM formation, remain limited. Most reported prevalence figures are derived from heterogeneous cavopulmonary populations and vary depending on patient selection, timing of evaluation, and imaging strategy [1,5-7].

Published Kawashima series predominantly describe late-onset venous collaterals or pulmonary arteriovenous malformations, typically presenting months to years after surgery [8-13]. As a result, early postoperative desaturation following an apparently satisfactory Kawashima repair is frequently attributed to pulmonary pathology, potentially delaying recognition of systemic venous decompression pathways.

The present report describes two infants with an interrupted IVC undergoing Kawashima operation who demonstrated divergent early postoperative oxygenation trajectories. By correlating clinical findings with targeted venographic assessment, this case series aims to highlight early systemic VVM formation as an under-recognized, hypothesis-generating mechanism of postoperative desaturation following Kawashima repair.

A preliminary version of this work was previously presented at the 8^th^ World Congress of Pediatric Cardiology and Cardiac Surgery (WCPCCS 2023 ), Washington D.C., August 27 - September 1, 2023 [14].

## Case presentation

Between November 2018 and November 2020, two patients underwent Kawashima operations at Bhanubhai and Madhuben Patel Cardiac Centre, Karamsad, Gujarat. The case report was approved by the Institutional Ethics Committee, H.M. Patel Centre for Medical Care and Education, Anand, vide Approval No. IEC/BU/2021/Cr.09/37/2021, dated 04.02.2021. Medical charts, echocardiography, cardiac catheterization (CC), cardiac computed tomography (CT), and operative notes were screened retrospectively. In our study, the data accumulated were classified into three different headings: 1) Pre-KO stage; 2) Peri-KO stage (Tables 1, 2); 3) Post-Kawashima Surgery Stage (Table 2).

**Table 1:** Patient data AP, antero-posterior; CC, cardiac catheterization; LPA, left pulmonary artery; MAPCAs, major aortopulmonary collateral arteries; RPA, right pulmonary artery; VSD, ventricular septal defect; SVC, superior vena cava; IVC, inferior vena cava; AV, atrio-ventricular; NA, not applicable

Sr. No.	Parameter	Case 1		Case 2
Demographic				
1	Age	1 year		1 year
2	Sex	Female		Female
3	Weight	6.5 kg		6.4 kg
4	Hemoglobin	12.3 g/dL		10.4 g/dL
Echocardiography				
5	Common atrium	Present	Present (large ASD)	
6	AV valve	Common AV valve	Common AV valve	
7	Ventricular morphology	Univentricular physiology	DILV / DORV	
8	VSD	Large	Large	
9	Pulmonary stenosis	Severe	Severe	
10	Pulmonary stenosis gradient	56 mmHg	NA	
11	Great artery relation	Malposed	Side-by-side	
12	Right PA size	14 mm	NA	
13	Left PA size	12 mm	Proximal stenosis	
14	Confluent PAs	Present	Present	
15	Heterotaxy	Present	Absent	
16	IVC anatomy	Interrupted, azygos continuation	Interrupted, azygos continuation	
17	McGoon ratio	2.50	1.566	
18	Nakata index	682.51 mm²/m²	396.12 mm²/m²	
19	MAPCAs	Absent	Present	
Computed Tomography (CT) findings				
20	CT performed	No		Yes
21	RPA proximal (pre-Kawashima)	NA		7.2 mm
22	RPA mid (pre-Kawashima)	NA		7.2 mm
23	RPA distal (pre-Kawashima)	NA		7.1 mm
24	LPA proximal (pre-Kawashima)	NA		2.6 mm
25	LPA mid (pre-Kawashima)	NA		4.4 mm
26	LPA distal (pre-Kawashima)	NA		9.5 mm
27	McGoon ratio (pre-Kawashima)	NA		1.566
28	Nakata index (pre-Kawashima)	NA		396.12 mm²/m²
29	RPA proximal (peri-Kawashima)	NA		8.0 mm
30	RPA mid (peri-Kawashima)	NA		7.6 mm
31	LPA proximal (peri-Kawashima)	NA		4.7 mm
32	LPA mid (peri-Kawashima)	NA		6.5 mm
33	LPA distal (peri-Kawashima)	NA		10.5 mm
34	McGoon ratio (peri-Kawashima)	NA		1.757
Cardiac Catheterization (CC) findings				
35	CC performed	No		Yes
36	Right PA filling	NA		Good
37	Left PA narrowing	NA		Focal, ~50%
38	Distal PA flow	NA		Good bilateral flow
39	Venous flow pattern	NA		SVC → IVC → SVC

**Table 2:** Peri-operative data CPB, cardiopulmonary bypass; ACC, aortic cross-clamp; POD, postoperative day

Sr. No.	Description	Case 1	Case 2
01	Pre-Bypass Pulmonary Artery (PA) Pressures	16/02 (12) mmHg	21/15 (17) mmHg
02	Cardiopulmonary Bypass Time (CPB)	76 min.	130 min.
03	Aortic Cross-Clamp (ACC) Time	Nil	Nil
04	Associated Procedures Performed	Patent Ductus Arteriosus (PDA) Ligation	PDA Ligation; Left Pulmonary Artery (LPA) Plasty
05	Ventilator Hours	103 Hours.	Indefinite
06	Length of Hospital Stay	15 days	Patient 2 expired on POD 22

Case 1

Pre-Kawashima Evaluation

**Figure 1:**
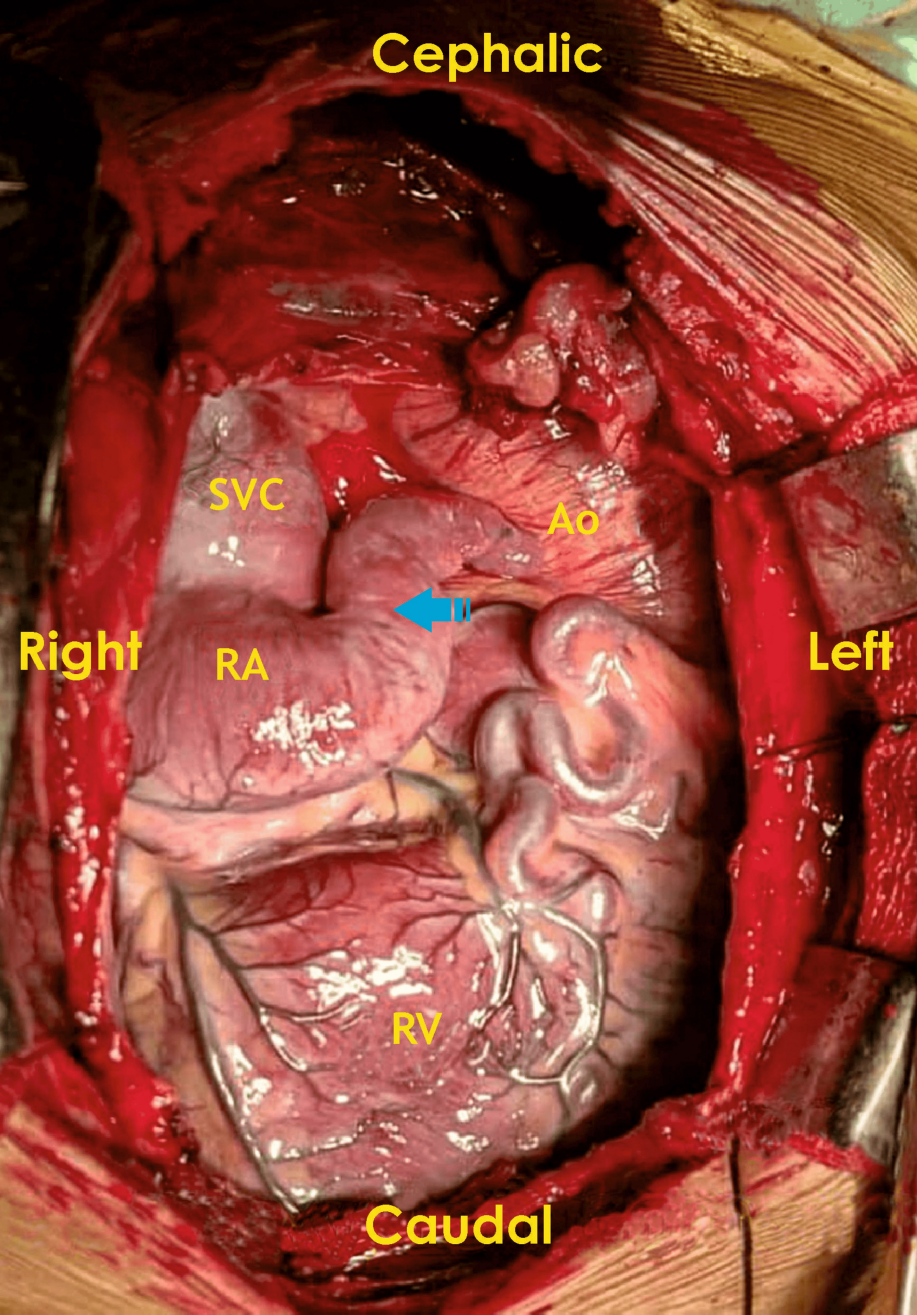
External anatomy of the heart in a patient with heterotaxy syndrome and left isomerism (Case 1) RA, right atrium; RV, right ventricle; SVC, superior vena cava; Ao, aorta The arrow indicates the region (left atrial appendage) demonstrating left isomerism and heterotaxy syndrome-related structural arrangement. Image Credits: Dr. Vishal V. Bhende

A 12-month-old female (weight 6.5 kg) presented with cyanosis and features of complex congenital heart disease. Hemoglobin was 12.3 g/dL. Transthoracic echocardiography demonstrated a common atrium, common atrioventricular valve, large ventricular septal defect resulting in univentricular physiology, severe pulmonary stenosis with a peak gradient of 56 mmHg, malposed great arteries, confluent branch pulmonary arteries (right pulmonary artery (RPA) 14 mm, left pulmonary artery (LPA) 12 mm), and heterotaxy syndrome. The inferior vena cava was interrupted with azygos continuation. The McGoon ratio was 2.50, and the Nakata index was 682.51 mm²/m². No pre-operative CT or catheter-based imaging was obtained (Figure 1).

Peri-operative Course

The patient underwent a Kawashima operation with patent ductus arteriosus ligation. Cardiopulmonary bypass time was 76 minutes, and no aortic cross-clamping was required. Pre-bypass pulmonary artery pressure was 16/2 (mean 12) mmHg. Postoperative ventilatory support was required for 103 hours. Total hospital stay was 15 days. Table 1 summarizes the detailed preoperative echocardiographic, computed tomography, and catheterization findings for both patients.

Post-operative Follow-Up

At discharge, resting room-air oxygen saturation (RASpO₂) was stable. Serial inpatient and outpatient pulse oximetry confirmed persistently stable room-air oxygen saturations, without clinical cyanosis or functional decline. In the absence of desaturation, echocardiographic evidence of unobstructed cavopulmonary pathways, and preserved systemic ventricular function, repeat cardiac catheterization was not clinically indicated, and the patient continues routine surveillance at the cardiac center. Table 2 provides an overview of intraoperative parameters, cardiopulmonary bypass details, and the early postoperative course.

Case 2

Pre-Kawashima Evaluation

**Figure 2:**
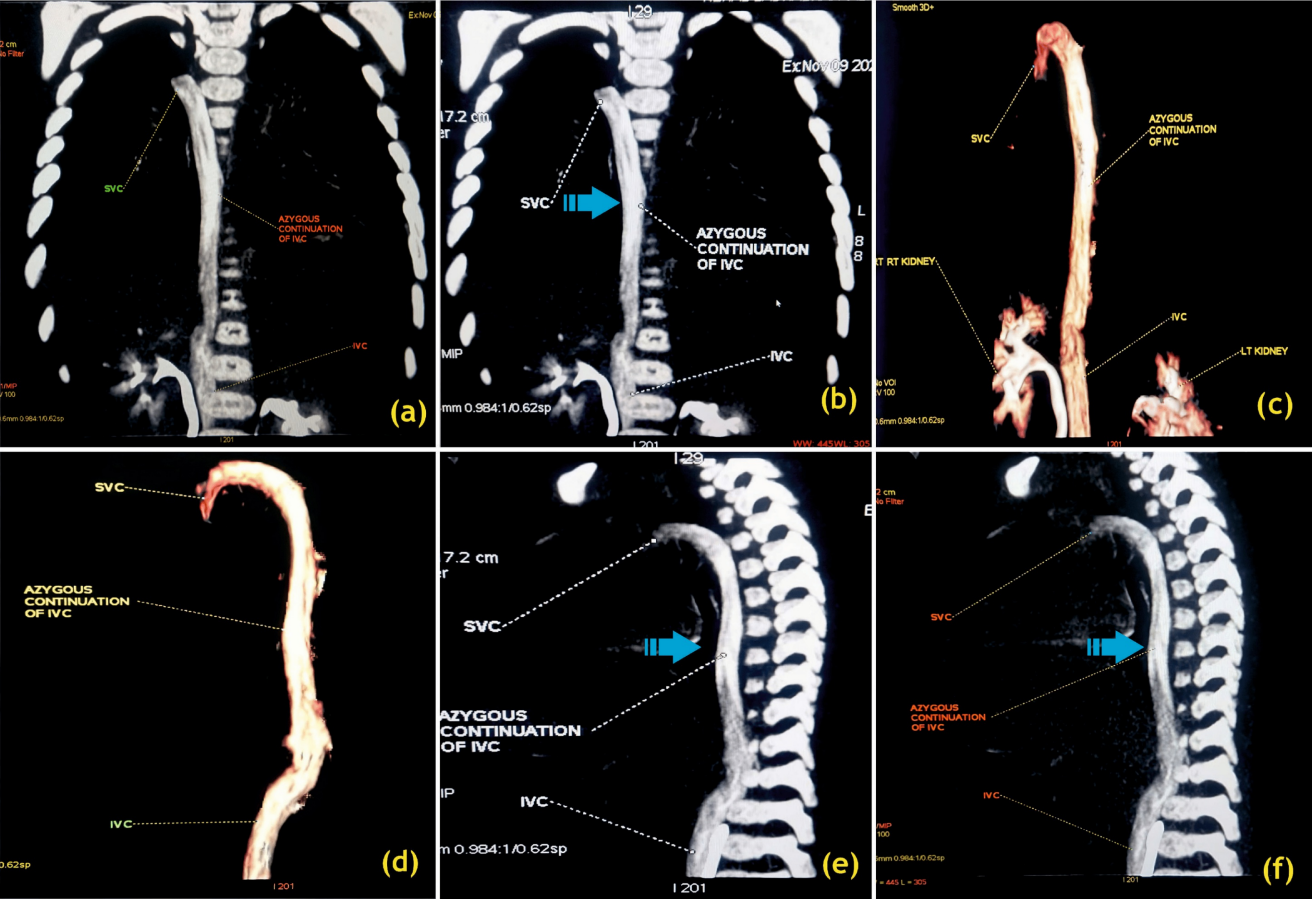
Azygous continuation in interrupted inferior vena cava (IVC) (A) MIP coronal image, (B) MIP coronal image, (c) VR coronal image, (D) VR sagittal image from the left side, (E) & (F) MIP sagittal image from the left side MIP, maximum intensity projection; IVC, inferior vena cava; LT, left; RT, right; SVC, superior vena cava; VR, volume rendering Arrows indicate the azygos continuation of the interrupted inferior vena cava. Image Credits: Dr. Deepakkumar V. Mehta

**Figure 3:**
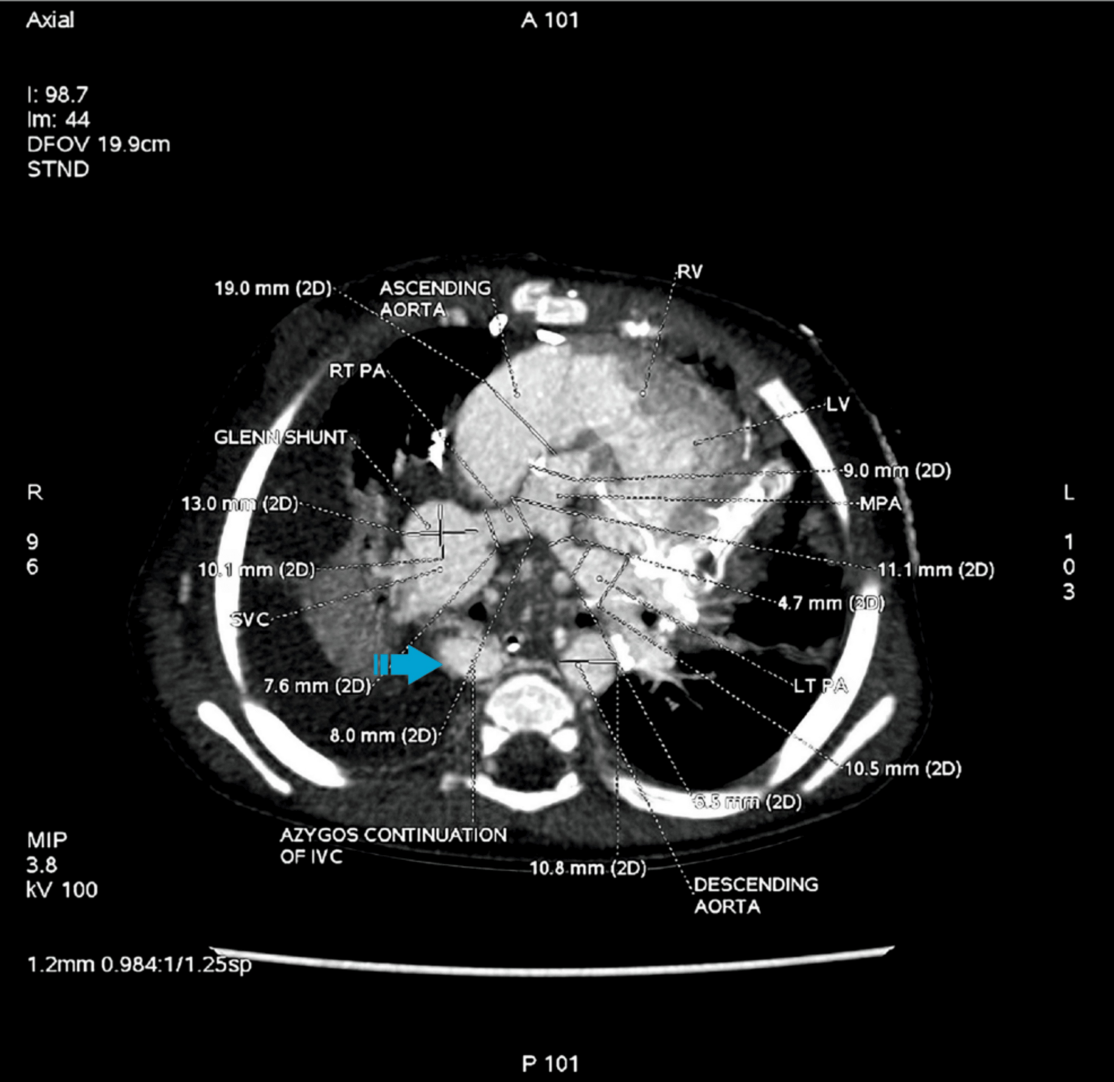
Contrast-enhanced axial maximum intensity projection (MIP) images of the thorax at the level of pulmonary arteries, showing azygos continuation of the IVC lying right anterolateral to the vertebral body IVC, inferior vena cava; LT, left; LV, left ventricle; MPA, main pulmonary artery; PA, pulmonary artery; RT, right; RV, right ventricle; SVC, superior vena cava The arrow highlights the azygos continuation seen adjacent to the vertebral body. Image Credits: Dr. Deepakkumar V. Mehta

**Figure 4:**
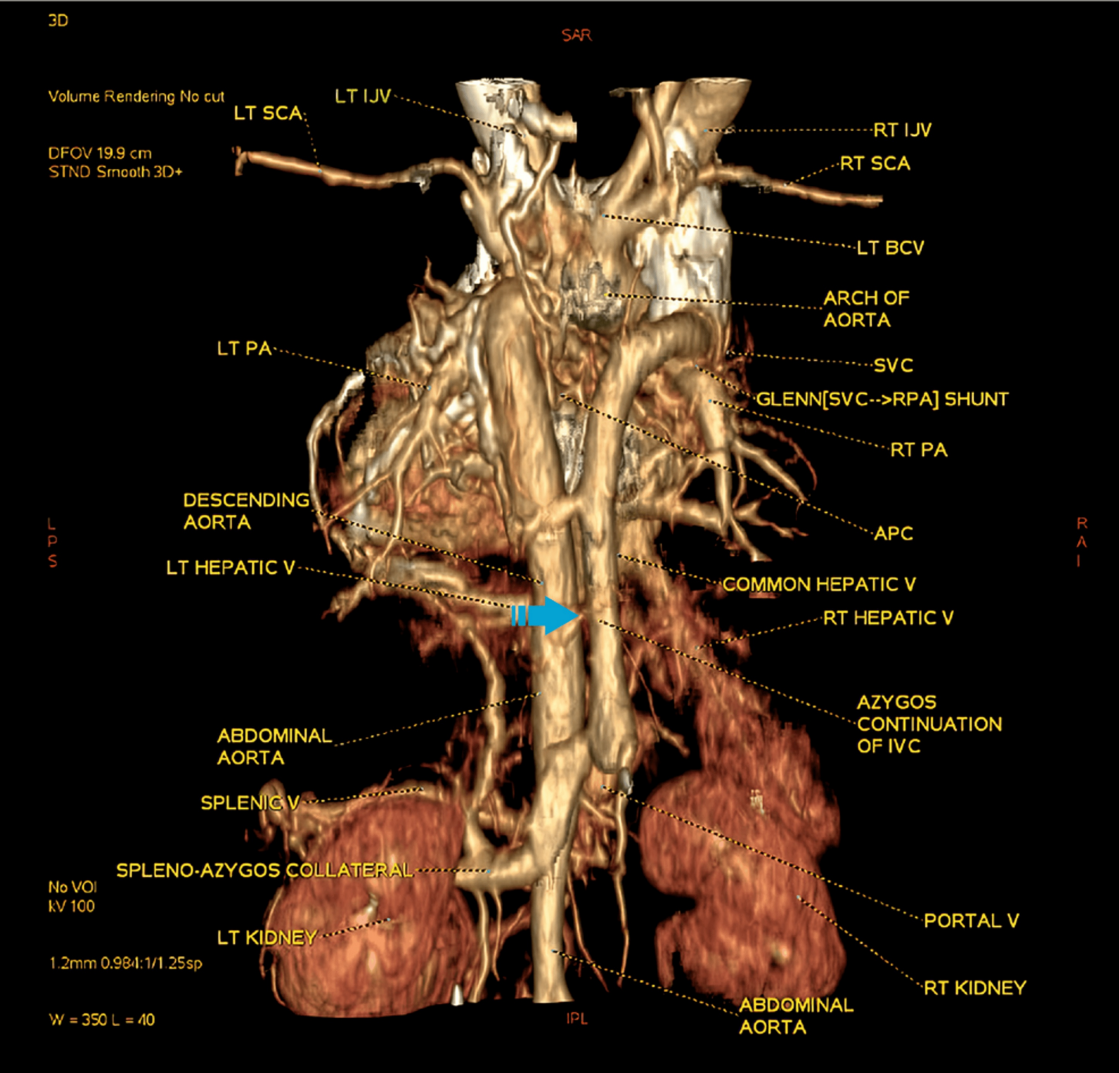
Image of a 3D volume-rendering (VR) posterior view displaying azygos continuation of the IVC lying on the right side of the descending thoracic aorta APC, aorto-pulmonary collateral; BCV, brachio-cephalic vein; LT.IJV, left internal jugular vein; LT.PA, left pulmonary artery; LT.SCA, left subclavian artery; RPA, right pulmonary artery; RT, right; SVC, superior vena cava The arrow demonstrates the azygos continuation as visualized in the posterior 3D rendering. Image Credits: Dr. Deepakkumar V. Mehta

A 13-month-old female (weight 6.4 kg) with hemoglobin of 10.4 g/dL presented with a double outlet right ventricle, double inlet left ventricle, large ventricular septal defect, severe pulmonary stenosis, common atrium, and interrupted inferior vena cava without heterotaxy. CT angiography revealed confluent branch pulmonary arteries with RPA diameters of 7.2-7.2-7.1 mm (proximal, mid, distal) and LPA diameters of 2.6-4.4-9.5 mm(proximal, mid, distal), giving a McGoon ratio of 1.566 and a Nakata index of 396.12 mm²/m². Major aortopulmonary collateral arteries were also noted (Figures 2-4).

Diagnostic cardiac catheterization showed good filling of the right pulmonary artery, focal 50% narrowing of the left pulmonary artery, and retrograde flow from the SVC into the IVC with subsequent return to the SVC, suggestive of early venous collateral formation.

Peri-operative Course

The patient underwent a Kawashima operation with concomitant patent ductus arteriosus ligation and left pulmonary artery plasty. Cardiopulmonary bypass time was 130 minutes, without aortic cross-clamping. Pre-bypass pulmonary artery pressure was 21/15 (mean 17) mmHg. Postoperative ventilatory support remained prolonged, and oxygen saturation declined significantly by postoperative day 9 (Tables 1, 2).

Post-operative Progress and Outcome

Progressive systemic oxygen desaturation occurred first on postoperative day 9, despite stable hemodynamics and adequate pulmonary artery flow. Pulmonary parenchymal pathology and pulmonary arteriovenous malformations were excluded by imaging, following which targeted venography demonstrated early systemic veno-venous malformations decompressing into systemic venous pathways, temporally corresponding with the onset of desaturation. The patient developed septic shock and succumbed on post-operative day 22 despite maximal supportive therapy (Table 2).

Post-Kawashima Surgery Stage

This consists of a sequential and prospective analysis of patient history, right atrial oxygen saturation (RASpO_2_), weight, hemoglobin values, chest X-ray, echocardiography, and CC. All individuals' resting state RASpO_2_ was measured during the most recent clinic follow-up. Echocardiographic analysis comprised ventricular and systemic atrioventricular valve function. CC data includes systemic ventricular end-diastolic pressure (EDP), pulmonary arterial pressure (PAP), pulmonary vascular resistance (PVR), and positive cavopulmonary (CP) gradient (Table 3).

**Table 3:** Diagnostic criteria for systemic oxygen desaturation based on AV or VVM PAVM, arteriovenous malformation; VVM, veno-venous malformation; AV, arteriovenous; SVC, superior vena cava; PA, pulmonary artery

Pulmonary AVM (PAVM)	Systemic VVM
A patient was diagnosed with PAVM if any of the three were present: Rapid PAVM transient (<3 heartbeats) of the contrast, which was introduced in the proximal PA angiography, Bubble echo analysis in the PA after agitated saline is directly injected. After the isotope was injected into a peripheral vein in the upper extremities, a lung perfusion scan (LPS) showed positive extra-pulmonary isotope use by the kidney or brain.	A venous channel known as systemic VVM allows blood to exit the pulmonary blood circulation and enter the atrium, pulmonary veins, or hepatic circulation. VVM is classified based on its destination, location, and size. Left SVC, right SVC, and large-diameter VVM were measured. The largest SVC's diameter was used to classify the VVM's caliber; less than 25% was classified as small, 25% to 50% as medium, and more than 50% as large. The abdominal azygous/hemizygous vein, innominate vein, femoral vein, and SVC are the four sites that were identified for injection to identify VVM.

Systemic oxygen desaturation (SOD) is nothing but a decrease in RASpO_2_ seen when the patient came for follow-up analysis (approximately ≥ 5%) following discharge after KO in the absence of any lung parenchymal conditions in X-ray.

**Figure 5:**
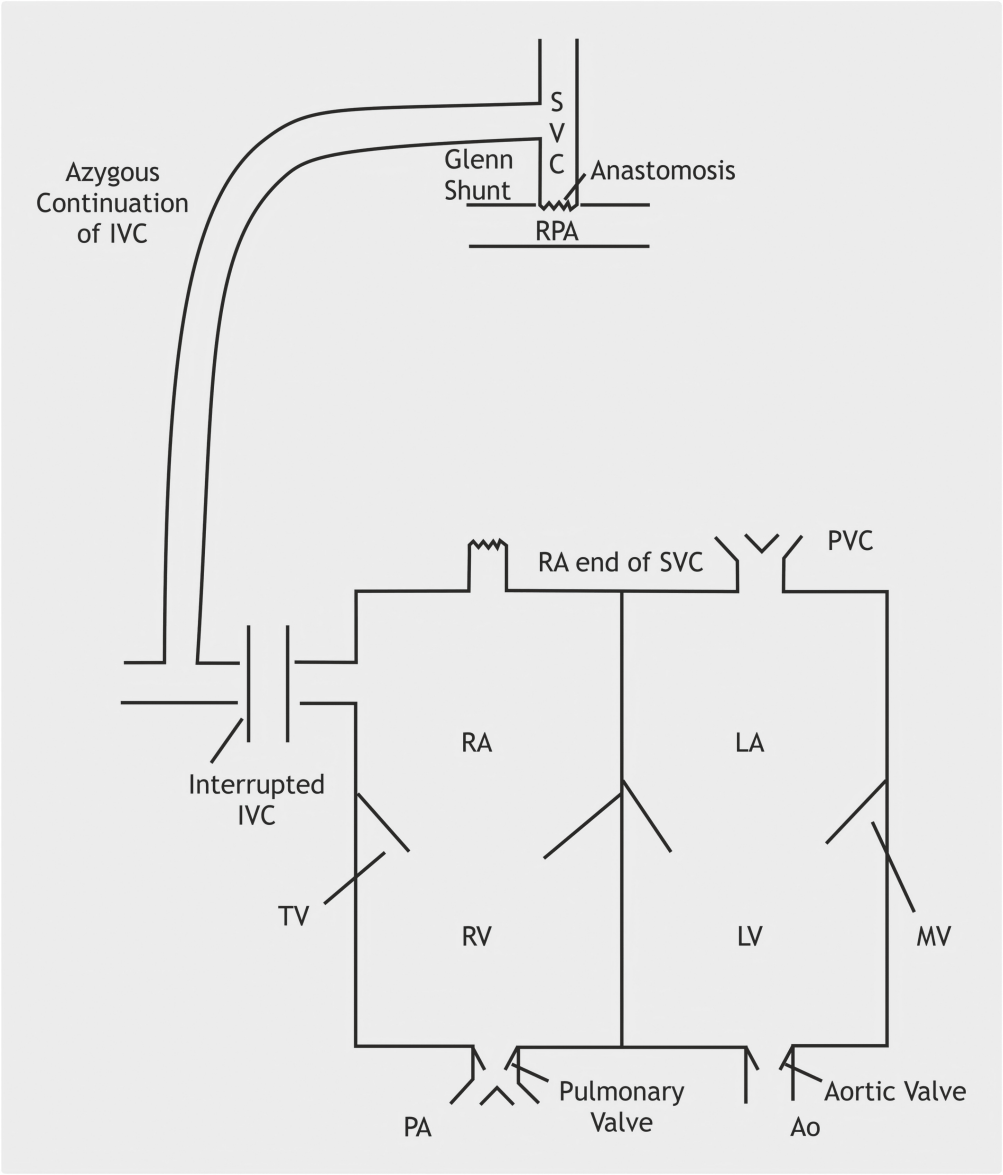
Graphical representation of Kawashima repair Ao, aorta; IVC, inferior vena cava; LA, left atrium; LV, left ventricle; MPA, main pulmonary artery; MV, mitral valve; PVC, pulmonary venous channel; RA, right atrium; RPA, right pulmonary artery; RV, right ventricle; SVC, superior vena cava; TV, tricuspid valve Image Credits: Dr. Vishal V. Bhende

These two cases illustrate the variable postoperative trajectory following the Kawashima operation (Figure 5). Although both patients had similar systemic venous anatomy and hepatic venous incorporation patterns, only one developed rapid postoperative VVM formation associated with profound systemic desaturation. The contrasting clinical courses highlight the need for rigorous postoperative surveillance and early imaging, particularly below-diaphragm angiography, when desaturation occurs without parenchymal lung disease or evidence of PAVMs.

This study represents a retrospective, descriptive case series. Inclusion was limited to patients undergoing the Kawashima operation during the study period, with complete perioperative, imaging, and follow-up data permitting a comparative assessment. Postoperative imaging and cardiac catheterization were clinician-triggered, based on unexplained desaturation or suspicion of venous decompression, rather than protocol-mandated screening.

## Discussion

In the present report, systemic oxygen desaturation occurred early, within the first postoperative week, with a clearly documented onset on postoperative day 9 in one patient, while the second patient maintained stable oxygen saturations throughout follow-up. This temporal relationship enabled focused venographic assessment and facilitated differentiation between pulmonary and systemic causes of desaturation, a distinction that is often challenging after the Kawashima operation [1,5].

When comprehensive angiography is performed above and below the diaphragm in patients with suspected venous decompression after cavopulmonary connections, systemic VVM can be identified with high diagnostic yield, as reported in selected observational series [1,3,5]. The detection rate cited in this context reflects case-based observations rather than systematic screening of all Kawashima patients.

Stumper et al. reported an 80% identification rate, while Kaneko et al. documented a 60% detection rate [1,3]. Failure to identify VVM preoperatively is possible when low inferior, or modified, venography was ignored as a regular diagnostic procedure.

VVM can present between 16 months and 6 years following KO. They can vary in size and number, potentially leading to clinically significant blood oxygen desaturation. Hepatic venous (HV) incorporation is one technique that has been reported to relieve post-Kawashima procedure desaturation.

We noted significant postoperative hemoglobin oxygen desaturation in arterial samples on day 9 in one patient, but no saturation reduction in the other patient, despite both patients having similar HV incorporation.

Several factors can account for the formation of venous collateral branches after KO: inherent venous anatomy and the difference in pressure gradient between the SVC and PA [7]. During embryonic IVC development, venous channels disappear, but then they may appear again when the pressure gradients increase in the vena cava following CP anastomosis. As such, VVM channels preoperatively remain undiagnosed but become obvious when the blood is rerouted, due to the production of decompression channels [8]. Nevertheless, it’s not explained why in some patients, VVM channels develop following KO, but don’t in the others. It is not so clear why the range of channel development differs so widely.

There are chances for VVM channel development to be related to the pressure difference between the two directional Glenn circuits, evidenced by the systemic ventricle, EDP, and PAP. Magee et al. found that the development of VVM was independently correlated with the level of gradient between the SVC and the PA [7]. In the current report, we found that both patients had no block at the anastomotic region and that they presented with a linkage between the SVC and the azygos system, which could allow the SVC venous blood to decompress and exit the pulmonary circulation (PC). Systemic desaturation is a particular problem related to VVM, which can occur regardless of whether VVM arises early or later. In our study, the second patient developed VVM on the ninth postoperative day; it should be considered because oxygen desaturation is a common side effect of Kawashima operations [9]. Considering hepatic vein redirection among patients with ideal hemodynamics for converting into a total CP anastomosis is ideal.

The proposed association between venous pressure gradients, embryologic venous channels, and recruitment of systemic VVM should be regarded as hypothesis-generating. Although prior studies have demonstrated correlations between CP pressure gradients and venous collateral formation, causality cannot be established from limited observational data [7].

In our two cases, hepatic veins were indirectly draining into the PC. Pulmonary AVMs were recorded in 21%-58% of individuals who underwent KO [10,11,12]. Patients who develop pulmonary AVM present like this in the first 30 months, with oxygen saturations dropping consistently and slowly into the high 70%. The reason for this is unknown; however, maldistribution and non-pulsatile PC and lack of hepatic factor because of a change in direction of normal HV blood flow from the PC are attributed to the pathogenesis [13].

Alternative explanations for early postoperative desaturation, including pulmonary parenchymal disease, airway pathology, ventricular dysfunction, and pulmonary arteriovenous malformations, were systematically evaluated and excluded using imaging, echocardiography, and clinical assessment [10-13].

This report is limited by its retrospective design, small sample size, and absence of routine pre- or post-Kawashima venographic screening. Serial documentation of VVM progression was not feasible in the patient with clinical deterioration. Consequently, these observations should not be extrapolated to incidence or causality but interpreted as hypothesis-generating, consistent with prior literature derived from heterogeneous cavopulmonary populations [1,5-7].

## Conclusions

Systemic veno-venous malformations may develop early after a Kawashima operation and can result in clinically significant oxygen desaturation even in the absence of pulmonary arteriovenous malformations. In patients with unexplained postoperative desaturation, selective targeted angiographic evaluation, including a below-diaphragm venous assessment, may aid in identifying systemic venous decompression pathways.

Given the observational nature of this report, routine angiographic screening for all patients cannot be recommended. However, focused evaluation in selected high-risk or desaturating patients appears justified. Early consideration of Fontan completion with hepatic venous incorporation may represent a preventive strategy against both pulmonary and systemic venous shunting, supported by existing literature, although prospective validation is required.
